# An Ultra-Low-Cost Optoacoustic Method for Imaging Specific Biological Structures

**DOI:** 10.3390/diagnostics16030436

**Published:** 2026-02-01

**Authors:** Sergio Contador, Álvaro Jiménez, Eduardo Lage, Carla López, Juan Aguirre

**Affiliations:** 1 Department of Electronic and Communications Technology, Medical Engineering Development and Innovation Center, Autonomous University of Madrid, 28049 Madrid, Spain; sergio.contador@uam.es (S.C.); alvaro.jimenezg@uam.es (Á.J.); eduardo.lage@uam.es (E.L.); carlalopezmtz@gmail.com (C.L.); 2 Health Research Institute of the Jiménez Díaz Foundation, 28040 Madrid, Spain

**Keywords:** optoacoustic imaging, single sensor, clinical imaging

## Abstract

**Background/Objectives:** Optoacoustic imaging technologies are emerging as promising tools for clinical practice. Several systems have the potential to fill specific niches in the medical imaging landscape thanks to a unique performance based on the combination of rich optical absorption contrast and high ultrasonic penetration-to-resolution ratios. However, current optoacoustic methods rely on tomographic reconstructions, which impose significant complexity on the systems in terms of number and distribution of transducers, acquisition electronics, and general operation. As a result, optoacoustic tomography apparatus are generally expensive and bulky and require intensive training for their operation. Here, we report on an optoacoustic imaging method that uses a single ultrasound transducer and non-tomographic image formation to overcome the drawbacks of classical tomographic methods. The method is designed for retrieving layered slab-like biological structures like tattoo ink, subcutaneous fat, muscles, or cerebrospinal fluid below the fontanelle. Moreover, it can be adapted to other geometries. **Methods:** We have implemented the method in a user-friendly, compact, simple, and low-cost system and tested its performance using simulations, synthetic phantoms, and biological phantoms containing tattoo ink. **Results:** Our results indicate that the system can discriminate slab-like structures from other shapes and recover them with the axial resolution of tomographic optoacoustic methods. The findings also suggest that the system has the potential to improve tattoo removal procedures. We further discuss its implications for pediatrics, traumatology, or endocrinology. **Conclusions:** This work paves the way for a new generation of simple, easy-to-use and low-cost imaging systems with the potential to impact several medical fields.

## 1. Introduction

Most medical imaging technologies are generally implemented in two different modes: tomographic and non-tomographic, each with their own clinical value. Tomographic implementations typically result in complex and expensive systems that require extensive training to operate. They rely on data acquired from different projections and on inverse problem algorithms to provide high-fidelity images. Non-tomographic approaches, in contrast, rely on a reduced number of projections and do not employ inverse problem algorithms/methods. These characteristics generally lead to cheaper and simpler user-friendly devices that offer lower imaging performance. Nevertheless, both implementations have distinct value in the clinical imaging space. For example, highly complex X-ray Computed Tomography systems coexist in hospitals with simple X-ray projection apparatus [1]. Similarly, advanced Single Photon Emission Computed Tomography systems share a room with Scintigraphy in nuclear medicine departments [2]. Last but not least, ultrasound systems can be operated in B-mode and C-mode, while A-mode also has its distinct clinical usefulness [3].

Optoacoustic imaging (OAI) is an emerging optical imaging method that combines the rich contrast of optical absorption with the high-resolution, deep-tissue imaging capabilities of ultrasound (US) [4,5]. The imaging process begins when low-energy optical pulses illuminate tissue. As light penetrates, it is selectively absorbed by chromophores causing a slight and localized temperature rise that leads to thermo-elastic expansion and the generation of ultrasound waves. These acoustic waves travel to the tissue surface, where their time profiles are recorded by multiple detectors. Finally, an image formation method converts these acoustic signals into 2D or 3D images, revealing regions of high optical absorption in deep tissue with the resolution of ultrasound methods.

Over the past 15 years, the optoacoustic field has blossomed, leading to a wealth of methods and systems, motivating the creation of several startup companies that aim to translate the technology from the lab to the clinic. Macroscopic and mesoscopic systems based on different geometrical configurations of transducer arrays have been proposed and extensively evaluated in clinical settings, demonstrating potential value for a wide range of applications across multiple medical specialties [6,7,8,9,10,11,12,13,14].

However, to the best of our knowledge, all the optoacoustic imaging methods (without considering optical resolution optoacoustic microcopy systems) are based on tomographic image formation approaches sharing the common drawbacks of other imaging modalities, i.e., system complexity, high cost, and extensive user training.

Optoacoustic tomography necessitates a large number of transducers with its associated complex and costly electronics [11,15,16]. When operating at higher frequencies (generally >20 MHz), a common approach is to scan a single element transducer. Under this configuration, the electronics complexity is largely reduced but the need for mechanical stages is introduced [17,18,19]. All tomographic approaches require, in any case, significant computational power to perform sufficiently fast reconstructions, adding cost and complexity to the systems. A single-sensor detector system with no scanning has been recently proposed [20] for lymph node detection. However, it only detects optoacoustic signals and does not provide imaging capabilities.

Here, we propose a non-tomographic optoacoustic method for imaging slab-like layered structures using a single ultrasound transducer without scanning. We have tested the method using simulations and phantom experiments. Moreover, we have implemented it in a low-cost and easy-to-use optoacoustic system.

The system has been tested for tattoo imaging in pig skin, demonstrating that it can image key parameters which are always unknown and play a major role in tattoo removal, which is a highly demanded procedure with high percentage of failure and harmful side-effects (see Section 4).

Many clinically relevant biological objects follow a slab-like layered structure. The fat layer, the melanin layer, and the cerebrospinal fluid (CSF) below the skull, just to mention a few. Therefore, the clinical relevance of the proposed method is promising for applications well beyond tattoo imaging and is discussed in this paper. Finally, we discuss how the method can be modified to image structures with other shapes, and we showcase how the cost can be straightforwardly reduced to few hundred euros, by switching from Nd:Yag laser for illumination to an overdriven laser diode.

## 2. Materials and Methods

### 2.1. The Non-Tomographic Imaging Method

The non-tomographic imaging method leverages three characteristics of optoacoustic signals: (1) The signal created by a slab consists of a plane wave traveling in the direction perpendicular to the slab surface [21]. (2) The width in time of the wave multiplied by the speed of sound (reciprocal distance) equals the width of the slab [21]. (3) If a planar detector is situated parallel to the slab surface, the wave will arrive in phase to the detector surface. As a result, each of the points of the detector will vibrate simultaneously, resulting in a constructive interference leading to a large signal.

Given the speckle free nature of optoacoustic signals [22,23], points 1, 2, and 3 will be fulfilled consistently as long as the detector is placed close enough to the slab (at sufficiently long distance, the plane wave will “degenerate” due to diffraction effects related to the finite height of the slab).

**Figure 1:**
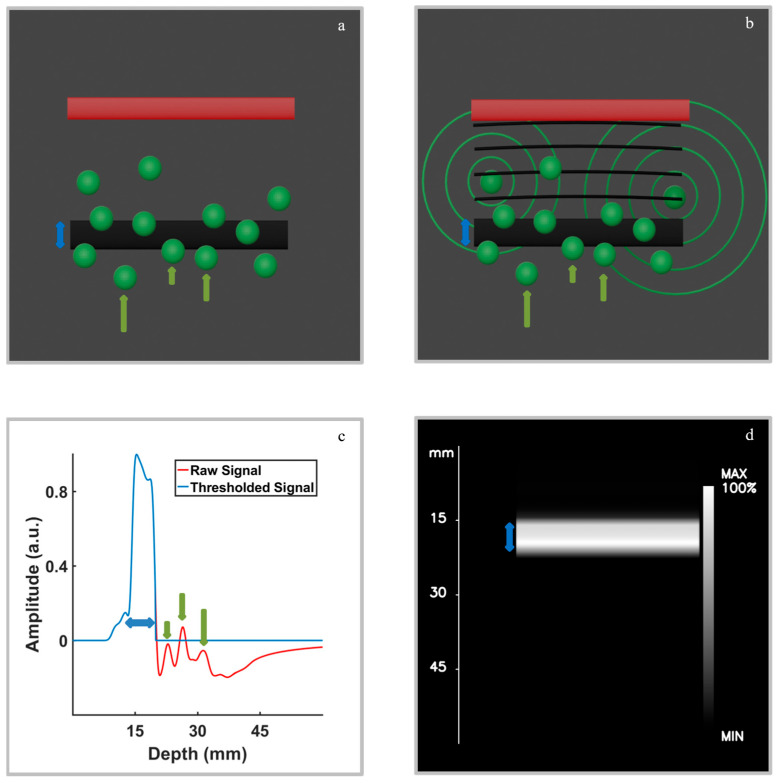
Illustration of the proposed non-tomographic optoacoustic imaging method and its ability to isolate signals from planar structures. (**a**) Schematic of the 2D simulation setup showing the flat absorber (black rectangle), background artifacts (green circles), and a flat ultrasound transducer (red block). The blue arrow represents the slab thickness, and the green arrows point to some of the spheres. (**b**) Conceptual illustration of acoustic wave propagation: the spherical waves emitted by the green artifact structures arrive at the transducer surface both in phase and out of phase, leading to constructive and destructive interference when integrated across all the surface positions, while the waves from the planar absorber arrive in phase, reinforcing the signal. (**c**) Raw (red) and thresholded (blue) optoacoustic signals. A distinct peak appears around 15 mm depth, corresponding to the planar absorber, with contributions from the artifacts minimized due to phase mismatch. The blue arrow indicates the signal region corresponding to the slab-like absorber, while the green arrows correspond to the remaining absorber signals. It should be noted that the optoacoustic signal results from the constructive and destructive interferences of the signals originating from all absorbers, not only those indicated with green arrows in (**a**). (**d**) Reconstructed non-tomographic image obtained from the thresholded signal in (**c**), highlighting the precise spatial localization of the planar structure (blue arrow).

The method is designed to retrieve planar structures (slab-like) whose surface runs parallel to the skin surface. It consists of several steps that result in the reconstruction of the slab, discriminating against the signal generated by any other object present in the field of view. First, a large planar detector (Figure 1a) is placed on the skin surface (i.e., parallel to the skin surface) together with an optical fiber arrangement for tissue illumination and subsequent optoacoustic signals generation. Given the three characteristics of optoacoustic propagation described above, any detected signal generated by a planar structure parallel to the skin surface will be significantly larger in comparison to the signal generated by any other structure (Figure 1b). Hence, a simple thresholding operation will eliminate any unwanted signal (Figure 1c). The resulting signal represents the z profile of the slab, which can be then represented in a 3D image from which a 2D maximum intensity projection can be obtained (Figure 1d).

Assuming that the slab area is much larger than the area of the detector, the reconstructed image shows the portion of the slab placed right below the detector, whose surface area equals the area of the detector (Figure 1d). The axial resolution (*δ*) of the method is given by the same parameters as the axial resolution of any planar optoacoustic imaging system, i.e.: *δ* = 0.88 $V_{s}/B$, where $V_{s}$ is the speed of sound and *B* the bandwidth [5].

### 2.2. Testing the Method with Simulations

The goal of the simulation study was to validate the underlying hypothesis of the proposed non-tomographic optoacoustic method, i.e.: the signals generated by non-planar objects can be discriminated against from the signals generated by slab-like objects by using a flat detector placed parallel to the slabs.

We performed 2D simulations to obtain the optoacoustic wavefront generated by a slab and many discs placed randomly around the slab. The simulations were run using the K-wave toolbox [24] in MATLAB (V2024) on a Hewlett Packard ProBook 640 G8 laptop (HP Inc., Palo Alto, CA, USA) equipped with a 4.2 GHz processor and 8 GB of RAM. A two-dimensional computational domain of 128 × 128 grid points was defined, with the origin at the top left corner and a spatial resolution of 0.5 mm in both the X and Y directions (field of view: 64 × 64 mm). The acoustic properties of the medium were set to mimic soft tissue, with a sound speed of 1500 m/s and a density of 1000 kg/$m^{3}$. A horizontally oriented slab was placed at the domain center. In index coordinates, the slab occupied rows 59–69 and columns 24–104 yielding a thickness of 11 grid points (5.5 mm) and a length of 81 grid points (40.5 mm). The slab was centered at (*X*, *Y*) = (31.75, 31.75) mm and bounded by ($X_{1}$, $X_{2}$) = (11.5, 52.0) mm and ($Y_{1}$, $Y_{2}$) = (29.0, 34.5) mm. A total of 30 circular artifacts (non-planar objects) with 2 mm diameter were added at random positions. The initial pressure distribution $p_{0}$ was defined as uniform (2 Pa) inside the absorbers (the slab and the circular artifacts), while the background was set to $p_{0}$ = 0.

Two sets of signals were then recorded: simulation 1a and simulation 1b. The signal of simulation 1a was recorded by a single element transducer placed at row 30 and column 64 occupied one grid point (0.5 mm) and centered at (*X*, *Y*) = (31.75, 14.75) mm. The signal of simulation 1b was recorded by a planar transducer located at row 30 spanning columns 29–99, yielding a thickness of one grid point (0.5 mm) and a length of 71 grid points (35.5 mm). The planar transducer was centered at (*X*, *Y*) = (31.75, 14.75) mm and bounded by ($X_{1}$, $X_{2}$) = (14.0, 49.5) mm and ($Y_{1}$, $Y_{2}$) = (14.5, 15.0) mm.

We then compared qualitatively simulation 1a and simulation 1b to observe the effect of the surface of the planar transducer on the signal (simulation 1b) against the single point signal (simulation 1a).

The non-tomographic image reconstruction algorithm was applied to the data from simulation 1b. The algorithm was implemented in Python as a two-stage pipeline comprising raw data processing and visualization. In the first stage, optoacoustic waveforms were converted from time to depth (*x*[mm] = 1.5 × *t*[µm]). Starting from the end of the waveform, the algorithm searched backward to identify the last sample exceeding 35% of the signal’s maximum value, which was defined as the threshold. A processed signal was constructed by setting all values beyond the threshold to zero, thereby isolating the portion of the waveform containing relevant features.

In the second stage, the processed signal was transformed into a 3D grayscale strip and rendered as an image. The 1D depth profile was broadcast across the horizontal dimension to generate a column-uniform intensity map. To improve visual continuity a region-aware Gaussian softening (blur *σ* = 2) was applied. The resulting image was min–max normalized to 8-bit (0–255) and rescaled by an automatically computed factor that limited saturation based on the component with the highest intensity.

### 2.3. Testing the Method with Experimental Phantoms

We wanted to further test the ability of the non-tomographic algorithm to discriminate the signal of planar structures from its surroundings in a 3D real phantom (experiment 1).

To acquire the necessary data, we developed a raster-scan optoacoustic mesoscopy (RSOM) system. As described further below, signals from a point-like detector and a planar detector can be derived from RSOM datasets.

The illumination source was a Q-switched DPSS laser (Onda NS; Bright Solutions Srl., Cura Carpignano, Italy) with an OEM laser head, controlled by a C-Box micro control unit (Bright Solutions Srl.). The laser emitted at 532 nm with pulse energy up to 1 mJ, pulse duration of 2–10 ns, and a repetition rate of 4 kHz. The beam was collimated by a 30 mm focal length lens (AC254-030-A; Thorlabs, Newton, NJ, USA) mounted on a CP33T/M cage plate (Thorlabs) and coupled into a multimode bifurcated fiber bundle (BF19Y2LS02; Thorlabs) consisting of 19 fibers with 200 µm core diameter, 320 µm coating diameter, and 2 m length. The bifurcated fiber ensured uniform illumination across the suture surface, thereby reducing potential shadows in the reconstructed images. For synchronization, a silicon (Si) photodiode (DET10A2; Thorlabs) was used to trigger the DAQ system.

Optoacoustic signals were detected by a single element high-frequency spherically focused ultrasound transducer (HFM23-PA-TR02-6V; Sonaxis, Besançon, France) with a focal length of 3 mm, a central frequency of 58 MHz, and 85% relative bandwidth, connected through a bias-tee (ZFBT-4R2GW; Mini-Circuits, Brooklyn, NY, USA). The transducer was integrated into a compact custom-made probe and co-aligned with the bifurcated fiber bundle to enable illumination and acoustic detection at the same region.

The probe was mounted on two motorized stages to implement scanning along two axes: a slow X-axis stage and a fast Y-axis stage. The slow axis was driven by a high-load linear stage with DC servo motor and ball-screw drive (M-414; Physik Instrumente, Karlsruhe, Germany), providing a 100 mm travel range and a maximum velocity of 100 mm/s, controlled by a C-863 Mercury servo controller (Physik Instrumente). The fast axis was driven by a precision linear stage with direct magnetic drive technology (V-408; Physik Instrumente). The probe was mounted on the fast stage, which in turn was mounted on the slow stage, with the two stages oriented orthogonally to provide perpendicular motion along the fast and slow scanning axes. This assembly was fixed to an aluminum breadboard.

For each capture, optoacoustic signals were digitized with a PicoScope 3406D oscilloscope (Pico Technology, St Neots, UK) at a sampling rate of 250 MS/s within a 7 µs acquisition window. The recorded waveforms were streamed to a laptop for processing and visualization using custom MATLAB and PYTHON routines.

We built a phantom (phantom 1) consisting of six non-absorbable polyamide monofilament sutures (Dafilon^®^, 6/0, DS9, 45 cm; B. Braun Melsungen AG, Melsungen, Germany) located around 1.5 mm above a strip of black tape (60 mm long, 16 mm wide). The sutures represent the artifacts (non-planar objects) and black tape the target slab. Sutures were secured with a custom holder consisting of a square frame with a side length of 60 mm and a thickness of 3 mm. Seven aligned holes were drilled on opposite sides of the frame to thread the sutures across. The suture ends were secured to the frame with adhesive. Each hole had a diameter of 1 mm, with 6 mm spacing between centers. The holes were numbered sequentially from 1 to 7 along both the upper and lower sides of the frame. Each pair of numbers denotes the upper and lower holes through which a suture was threaded to determine its orientation and intersection within the frame. Using this scheme, the desired pattern was generated by arranging the sutures between the following upper-to-lower hole pairs: 2–7, 3–6, 4–4, 5–5, 6–2, and 7–3. This configuration produced a pattern in which some sutures were parallel, while others intersected at different levels, mimicking location of potential artifacts in biological tissue (capillaries).

The black tape was attached to the inner surface of a square methacrylate tank which was filled with water to serve as the acoustic coupling medium. The suture holder was placed on top of the black tape and secured to the base of the tank. The region of interest (ROI) selected, measuring 6 × 6 mm to match the active area of the transducer, contained both sutures and black tape. The tank was coupled to a manual translation stage XYT1/M (Thorlabs), allowing fine adjustment of the distance between the phantom and the probe through a travel micrometer.

To validate that the signals generated by non-planar objects can be discriminated against the signals generated by planar objects, we acquired an RSOM dataset from phantom 1 (real data 1). The raster scan covered 6 × 6 mm field of view and comprised 2500 samples per A-line and 4000 fast axis acquisitions (one B-plane consisting of 4000 A-lines and 1 × $10^{7}$ samples) and 200 slow axis acquisitions (200 B-planes, 8 × $10^{5}$ A-lines, 2 × $10^{9}$ samples). The spatial sampling was 1.5 µm along the fast axis and 30 µm along the slow axis. The sutures were placed around 500 ± 200 µm below the focal point of the transducer.

From the RSOM dataset, we could compare the signal from a point-like detector against the signal from a planar detector as follows: On one hand, since in RSOM, each transducer focal point position is equivalent to a virtual point like detector, the signal corresponding to the point-like detector was obtained as the A-line from the central transducer position (real 1a); on the other hand, the signal corresponding to a virtual planar detector can be obtained by summing up all the A-lines in an area that would correspond to the detection area of the planar detector (real 1b). Both signals were represented for comparison.

Last but not least, we performed the non-tomographic reconstruction corresponding to the virtual planar detector (real 1b) and compared it to the RSOM tomographic reconstruction of the whole RSOM dataset (real data 1), which was performed using frequency domain inversion [25]. The non-tomographic image reconstruction algorithm described in previous section was applied to the experimental data real 1b with the following modifications. First, the Hilbert transform was applied to the A-line. After depth conversion, the dataset was corrected for baseline drift by subtracting the DC component. Once the thresholded signal was calculated (threshold = 27%), a step weighting function was applied to account for depth-dependent light attenuation.

### 2.4. Implementation of the Imaging Method on a System

We implemented the imaging method in a system, and we tested its imaging performance with a focus on tattoo imaging.

The system comprises four primary components: (1) optical excitation subsystem; (2) handheld detection probe; (3) DAQ unit for data acquisition; and (4) laptop for data processing and image visualization.

The optical source was a Cobolt Tor XS Q-switched laser (HÜBNER Photonics, Solna, Sweden) emitting at 1064 nm with pulse energy of 50–100 µJ and pulse duration of 1.5–3.5 ns operating at 1 KHz, suitable for generating optoacoustic signals in tissue. The laser was mounted on a Cobolt HS-03 heat sink (HÜBNER Photonics) and controlled via a dedicated Cobolt CE/CDRH control box (HÜBNER Photonics). A 30 mm focal length lens AC254-030-A (Thorlabs) mounted on a CP33T/M cage plate (Thorlabs) was used to collimate the beam and couple it to an optical fiber, which will deliver the beam to the tissue. We used a multimode optical fiber FP1000ERT (Thorlabs) with 1 mm core diameter, 1.4 mm coating diameter, and 1 m length. A Si photodiode (DET10A2; Thorlabs) was used to synchronize the DAQ with a nanosecond trigger signal (1–5 V).

The optoacoustic signals were detected by a single element flat piezoelectric transducer V324-SM (Olympus NDT; Waltham, MA, USA), with a central frequency of 25 MHz, 56% relative bandwidth, and a nominal active element diameter of 6 mm, coupled with a 5682 preamplifier (Olympus NDT) used to amplify the received signal before digitization. The transducer was integrated into a compact handheld probe, co-aligned with the optical fiber to enable simultaneous illumination and detection at the same area.

Signal acquisition was carried out using a PicoScope 3406D oscilloscope (Pico Technology), sampling each A-line at 500 MS/s over a 7 µs window, sufficient to resolve depth-dependent signal features with sub-millimeter resolution. All acquired data was transferred to a laptop, which performs the subsequent signal processing and visualization using custom scripts developed in MATLAB and PYTHON.

### 2.5. Depth Resolving Capabilities of the System: A Synthetic Phantom Experiment

To validate the depth-resolving capability of the system and get a grasp on the possible diffraction effects on the performance of the algorithm, a phantom-based (phantom 2) depth detection experiment was performed (experiment 2).

Phantom 2 consisted of a mixture of 1 g agar-agar (E406; Laguihoat, Madrid, Spain), 0.006 mL of black India ink (no. 951; Windsor & Newton, London, England), and 31.25 mL of water, simulating a tattoo. The mixture was poured into a 70 × 70 × 5 mm mold containing a 60 × 60 × 3 mm cavity and subsequently dried, resulting in a phantom with dimensions identical to those of the cavity.

The probe was mounted on a two-axis linear translation stage (XYT1/M; Thorlabs) with a 13 mm travel range, positioned above the water-filled tank where the phantom was immersed. Sub-millimeter adjustments of the distance between the phantom and probe were achieved using the travel micrometer. The stage was vertically attached to an aluminum breadboard (MB4560/M; Thorlabs) that was in turn mounted on an ultralight series II optical breadboard (PBG52514; Thorlabs) using three VB01A/M vertical brackets (Thorlabs).

Optoacoustic signals were acquired in 0.2 mm increments up to 2 mm from an initial position calculated to ensure a safe movement margin between the probe, phantom, and motor. After fixing the initial setup, the first measurement and micrometer screw reading were recorded. The signal’s global minimum was taken as the system distance position and the screw reading as the real distance. The micrometer screw was then raised by 0.2 mm for each subsequent measurement, repeating the process until it covered 2 mm in total. This yielded 22 measurements: 11 from the micrometer screw and 11 from the system.

Statistical analysis was used to validate depth estimates via ordinary least squares regression of real depth versus system depth. Two-sided *p*-value and the coefficient of determination *R* were computed to demonstrate a statistically significant linear association between measures and to quantify goodness of fit.

The possible diffraction effects were evaluated accounting for changes in the shape of the signal as a function of depth.

### 2.6. Imaging Tattoo in Pig Skin

We wanted to demonstrate the potential of the system for imaging relevant tattoo parameters: the tattoo depth, defined as the distance from the skin surface to the top part of the tattoo, and the axial thickness, defined as the axial extent of the tattoo pigments.

While the capability of the system to image tattoo depth is well characterized in experiment 2, we proceeded to test the system’s ability to resolve the axial thickness of different tattoos created in ex vivo pig skin (experiment 3).

Three fresh pork belly samples obtained in supermarket were prepared. Each sample was tattooed with three circular marks of 1 cm diameter, with an estimated axial thickness close to 1 mm, 2 mm, and 3 mm. A professional tattoo artist performed the procedure using an SRM 9-needle open-linear cartridge (0.40 mm; Cheyenne, Berlin, Germany) and Triple Black tattoo ink (Dynamic Color, FL, USA). After completing the tattooing procedure, three signal datasets were recorded for each pork belly sample using the system described in Section 2.4.

Following data acquisition, we estimated the axial thickness of each tattoo using the non-tomographic image reconstruction algorithm. To that end, we used the same two-stage pipeline described in Section 2.2, with the following modifications. Raw datasets were corrected for baseline drift by subtracting the DC component and Hilbert enveloped. A depth-dependent threshold of 7% was applied to the signal. A multiplicative step function was applied to correct for the decrease in light absorption with depth, ultrasound absorption, and the mismatch between the size of the tattoo layers and the frequency range of the transducer.

The axial thicknesses obtained by the optoacoustic system were compared to the axial thicknesses obtained by a gold standard method. The gold standard estimation was performed slicing the tattooed skin regions perpendicularly to the skin surface and taking photographs with a smartphone including a millimeter grid positioned beneath the sample to determine the pixel size. After pixel size calibration, tattoo axial thickness was measured from the skin surface to the deepest visible boundary of the pigment layer by placing two vertically aligned points (surface and bottom) on the image. The vertical separation between these points provided the axial thickness in millimeters.

Statistical analysis was performed to validate axial thicknesses measurements obtained from the cross-sectional photographs against those estimated by the non-tomographic optoacoustic reconstruction algorithm. Least squares regression was conducted using the manual measured pigment axial thickness and the values estimated with the system. Two-sided *p*-value and the coefficient of determination *R* were calculated to assess goodness of the fit and its statistical significance.

## 3. Results

### 3.1. Testing the Method with Simulations and Experimental Phantoms

**Figure 2:**
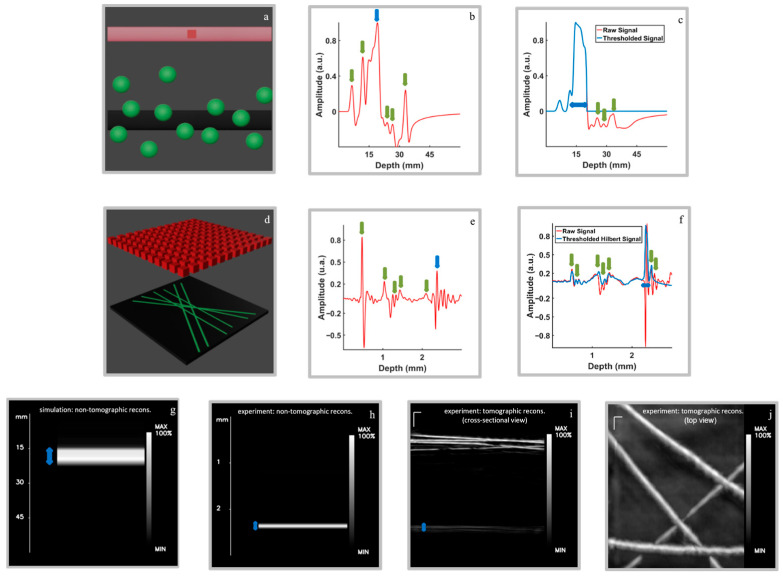
Schematic representations, raw optoacoustic signals, and reconstructed depth-resolved images from both simulated and experimental data. Panels (**a**–**c**): two-dimensional numerical simulations performed using K-wave toolbox in MATLAB. (**a**) Schematic of the 2D simulation domain, showing the slab (black rectangle), artifacts (green circles), and the transducer elements (red squares). (**b**) Raw optoacoustic signal acquired from simulation 1a, where the slab and artifact contributions to the signal overlap. The blue arrow indicates the region where the slab-like absorber is located, and the green arrows point to some of the spheres (absorbers). (**c**) Raw (red) and thresholded (blue) optoacoustic signals from simulation 1b, where the slab (peak around 15 mm depth) is clearly distinguishable from the artifacts. The blue arrow represents the slab axial thickness while the green arrows point to the lowered peaks of the absorbers. Panels (**d**–**f**): Experimental results obtained from custom-built 3D phantom. (**d**) Schematic representation of the 3D phantom setup, illustrating the volumetric distribution of the slab (black tape), artifacts (sutures), and the virtual point-like positions of the transducer on the acquisition grid (red cubes). (**e**) Raw optoacoustic signal acquired from real 1a, with overlapping contributions from the slab (blue arrow) and the artifacts (green arrows). (**f**) Raw (red) and thresholded Hilbert (blue) optoacoustic signals from real 1b, where the slab (peak around 2.3 mm depth) is clearly distinguishable from the artifacts. Again, the blue arrow indicates the region where the slab-like absorber is located, while the green arrows correspond to the remaining absorbers. Panels (**g**,**h**): non-tomographic reconstruction images derived from simulation 1b (**g**) and real 1b (**h**), highlighting the spatial distribution of the slab. Panels (**i**,**j**): tomographic image reconstruction from the whole RSOM dataset (real data 1), showing a cross-sectional view (**i**) and top view (**j**) of the slab and artifacts. Scale bars = 300 μm.

Figure 2a–c show the results of simulation 1, designed to evaluate the ability of the proposed approach to distinguish a planar absorber from the artifacts. The simulation domain (Figure 2a) includes a black planar slab, several spherical absorbers acting as artifacts, and the transducer positioned above them. When using a single transducer element (simulation 1a, Figure 2b), the recorded signal contains multiple peaks of similar amplitude, originating from both the planar and non-planar absorbers. In this configuration, the contributions from the slab and the artifacts overlap, making it impossible to distinguish the planar structure. In contrast, when a planar transducer is used (simulation 1b, Figure 2c), a single dominant peak clearly appears at the position corresponding to the slab (peak around 15 mm depth), while smaller peaks approximately one order of magnitude weaker represent the residual contributions from the non-planar absorbers (several peaks around 30 mm depth). These can be effectively removed by applying the thresholding procedure.

Figure 2d–f present the results of experiment 1, which validate the findings obtained from the simulations. The schematic representation of the experimental setup is shown in Figure 2d, illustrating the volumetric distribution of the slab (black tape), the absorbers (sutures) acting as artifacts, and the array of virtual point-like detectors. The signal recorded by the point-like detector (real 1a, Figure 2e) exhibits a large peak around 0.5 mm depth and two peaks of comparable amplitude, resulting from overlapping contributions of the slab and the artifacts. As in the simulated case, this configuration prevents clear discrimination of the slab structure. When the planar transducer is employed (real 1b, Figure 2f), a dominant peak emerges at approximately 2.3 mm depth, corresponding to the slab. Smaller peaks (around 2.5 mm depth) are associated with the surrounding absorbers and can be removed through the thresholding procedure.

The corresponding non-tomographic reconstruction of the signals from simulation 1b and real 1b are shown in Figure 2g and Figure 2h, respectively. The images confirm the accurate spatial localization of the planar absorber in both cases, demonstrating that the planar transducer enables clear discrimination between the pigment and the artifacts within the simulated and experimental domain.

Figure 2i and Figure 2j show the cross-sectional and top views of the tomographic reconstruction obtained from real data 1, respectively. In the cross-sectional view (Figure 2i), the suture region can be identified in the upper part of the image, while the pigment slab appears beneath it, corresponding to the same depth observed in the non-tomographic reconstruction. In the top view (Figure 2j), the sutures are clearly resolved, displaying sharp spatial definition and good contrast relative to the surrounding regions, whereas the black tape is visible in the background with lower contrast. Note that in both figures, only four out of six sutures are displayed, as the selected ROI includes only these four sutures. These results confirm that the tomographic reconstruction accurately preserves the spatial arrangement of both the sutures and the pigment layer, further validating the depth localization achieved by the proposed approach.

### 3.2. Implementation of the Method in an Imaging System and Depth Experiment

**Figure 3:**
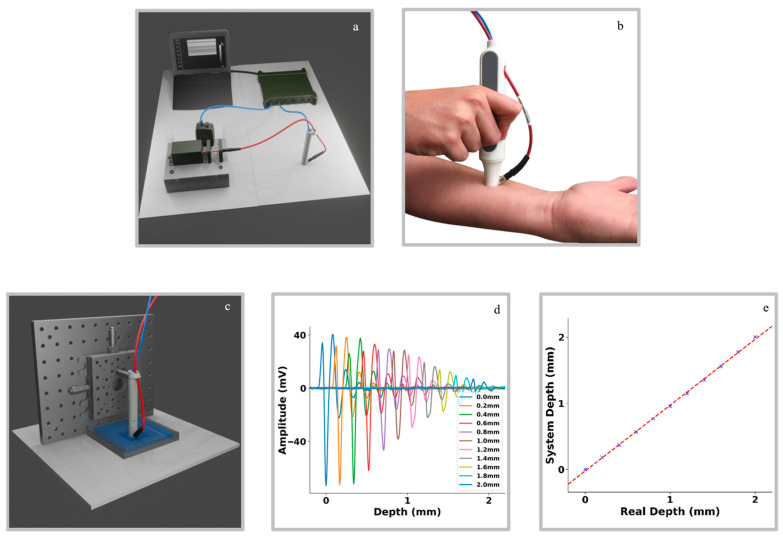
Optoacoustic system setup and demonstration of depth detection capability. (**a**) Schematic representation of the custom-built optoacoustic system implemented to assist tattoo removal applications. The setup consists of four main components: the optical subsystem (bottom left), the handheld probe assembly (bottom right), the DAQ unit (top right), and a laptop (top left) processing the incoming data. (**b**) Photograph of the handheld probe, illustrating the integrated ultrasound transducer and the securely mounted optical fiber (in red) for illumination and detection. (**c**) Schematic illustration of experiment 2. The handheld probe is clamped in a custom holder mounted to a manual translation stage, allowing precise vertical adjustment of the distance between the probe and a water-filled tank containing the phantom. (**d**) Results of experiment 2. Optoacoustic signals obtained as a function of the distance between the transducer and the slab. Measurements of the distance between the phantom and the probed were acquired at depths incremented in 0.2 mm steps from the initial position to 2 mm beyond using the micrometer screw. Each depth is represented by a different color (see legend). (**e**) Quantitative comparison between real depths and the corresponding measurements obtained by the optoacoustic system. Each blue marker represents an individual measurement, while the red dashed line indicates the values of a straight line fitted to the results using ordinary least squares linear regression.

In Figure 3a, we show a scheme of all the components of the imaging system, including the optical subsystem, the handheld probe assembly, the DAQ unit, and the processing laptop. Figure 3b shows a real picture of the handheld probe placed on the arm of a volunteer, integrating the ultrasound transducer and the illumination fiber. The setup for experiment 2, where the probe is fixed on a manual translation stage above a water tank containing the phantom, is illustrated in Figure 3c.

The results of experiment 2 are shown in Figure 3d and Figure 3e, respectively. The raw optoacoustic waveform plot as a function of time clearly shows a systematic shift in the main peak of each measurement with increasing depth (Figure 3d), confirming the sensitivity of the system to sub-millimeter depth changes. A quantitative comparison between the measurements taken with the micrometer screw and those measured by the system (Figure 3e) reveals an excellent agreement, with a Pearson correlation coefficient of *R* = 0.9996 (*p* = 1.876 × $10^{-15}$). These results demonstrate that the optoacoustic system can accurately retrieve depth information, supporting its use for assessing tattoo depth.

Regarding the possible diffraction effects on the shape of signal, it can be noted that the shape of the signal barely changes with depth. A small change in the peak after one millimeter is observed, but it does not affect the depth measurement. Such a change can be attributed both to diffraction effects and the selective absorption of high frequencies by tissue.

### 3.3. Imaging Tattoos in Pig Skin

**Figure 4:**
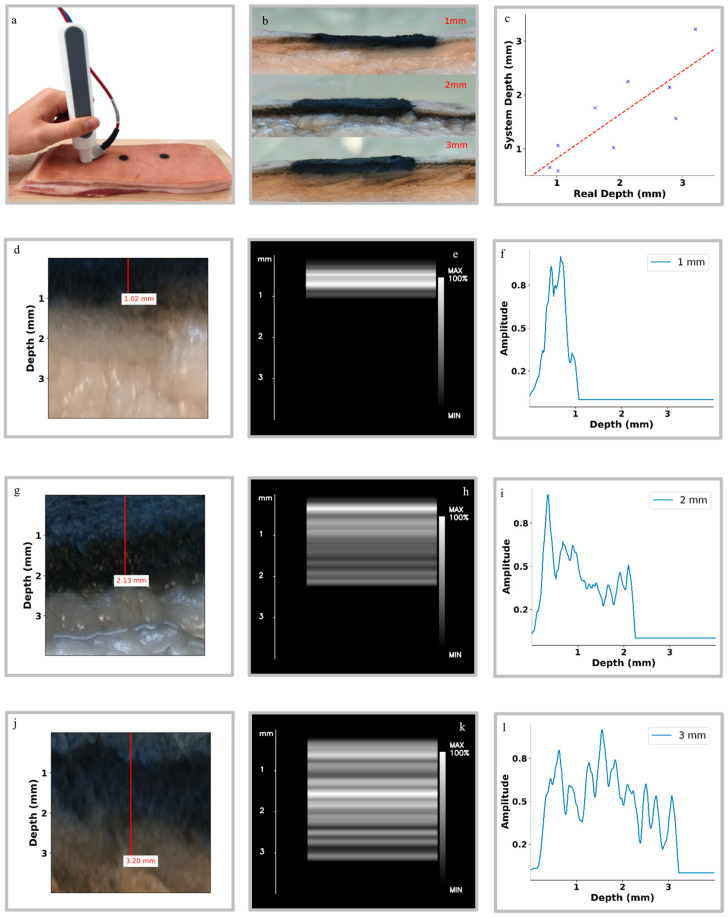
Experimental results from optoacoustic detection of tattoo pigments with different axial thicknesses in ex vivo pork belly skin. (**a**) Photograph of the experimental setup showing the handheld optoacoustic probe positioned over the tattooed pork belly phantom. (**b**) Cross-sectional view of the phantom, revealing three distinct pigment insertions corresponding to tattoo axial thicknesses of 1 mm, 2 mm, and 3 mm. (**c**) Quantitative comparison between the gold standard axial thicknesses and the corresponding measurements obtained by the optoacoustic system. Each blue marker represents an individual measurement. System measurements were fitted to a straight line (red) using ordinary least-squares linear regression. Panels (**d**,**g**,**j**): Photographs of the sliced pork belly tissue showing zoomed views of the tattoo pigment located at 1.02 mm, 2.13 mm, and 3.20 mm, respectively, as measured manually. Panels (**e**,**h**,**k**): non-tomographic reconstructed optoacoustic images corresponding to the pigment axial thicknesses shown in (**d**,**g**,**j**), respectively, obtained using the optoacoustic system. Panels (**f**,**i**,**l**): processed Hilbert envelopes derived from the optoacoustic signals at the three respective axial thicknesses processed using the non-tomographic reconstruction method. These signals confirm the system’s ability to detect pigments at varying axial thicknesses in skin.

The results of experiment 3 are shown in Figure 4. Figure 4a displays one of the three fresh pork belly samples prepared with three circular markings indicating tattoos of 1 mm, 2 mm, and 3 mm axial thickness, respectively. The handheld optoacoustic probe is positioned on the surface of the sample, illustrating the measurement procedure for the first tattoo layer. Figure 4b shows cross-sectional views of the three tattooed regions, revealing evident variations in pigment quantity and spatial distribution associated with the different axial thicknesses. Figure 4d and Figure 4g, and Figure 4j show photographs of the sliced pork belly tissue at each corresponding tattoo, with zoomed views and a vertical red line indicating the manual measured pigment axial thickness yielding axial thicknesses of 1.02 mm, 2.13 mm, and 3.20 mm for the 1 mm, 2 mm, and 3 mm tattoos, respectively.

The non-tomographic reconstructed optoacoustic images and their corresponding Hilbert envelopes are shown in Figure 4e and Figure 4h, and Figure 4k, and Figure 4f and Figure 4i, and Figure 4l, respectively. In the reconstructed images, the tattoo pigment appears as a bright, well-defined band whose width increases with tattoo thickness. For each case, the depth location of this band matches the real pigment axial thickness obtained from the photographs (Figure 4d,g,j) and indicated by the vertical red markers, confirming that the non-tomographic reconstruction provides a good estimate of the actual pigment depth. A statistically significant linear correlation (Figure 4c) is observed between the gold standard measured axial thickness and the axial thickness estimated by the system across the nine cases (*R* = 0.8254, *p* = 6.149 × $10^{-3}$), demonstrating the ability of the proposed optoacoustic imaging method to quantify tattoo pigment axial thickness. The results are also consistent across the three independent samples, suggesting good reproducibility of the measurements.

## 4. Discussion

We have proposed a non-tomographic optoacoustic imaging method that enables high-axial-resolution visualization of slab-like structures within tissue. We have demonstrated the method with simulations, as well as with synthetic and biological phantoms.

Our results with ink in a synthetic phantom and in biological phantoms (pig skin) indicate that the system has strong potential to measure the depth of tattoos. Tattoo removal procedures based on selective photo-thermolysis could greatly benefit from this imaging capability. By measuring the depth of the tattoo with micrometer resolution, dermatologists can accurately assess the evolution of the tattoo after each laser session in order to prescribe additional sessions or terminate the treatment; furthermore, the laser spot size can be optimized [26]. Moreover, by analyzing the ratio between the optoacoustic signal in the melanin layer and the tattoo ink, the wavelength that maximizes the amount of light absorbed by the ink in comparison with the amount of light absorbed by the melanin can be calculated.

The axial thickness of tattoos could also be measured, but with less accuracy than their depth. This is for two reasons. First, tattoo axial thickness may be underestimated if the concentration of the ink at the upper layers is too high, absorbing all the available laser energy. Second, the frequency range in which the transducer operates (5 MHz to 40 MHz) is not fully appropriate to image axial thickness values of 1–3 mm. Nevertheless, the layered nature of the tattoo and the custom-step function depth correction allowed us to measure the axial thickness with an accuracy of *R* = 0.8254 and *p* = 6.149 × $10^{-3}$. Choosing transducers with higher bandwidth or lower central frequency would alleviate this problem.

The potential clinical impact of the system extends well beyond tattoo removal. For example, the fat below the dermis typically forms a distinct layer and could be imaged using an appropriate excitation wavelength [27]. Imaging the axial thickness of the fat layer could be useful for the diagnosis of lipedema [28]. Similarly, the CSF below the fontanelle in neonates is contained in a slab-like layer (the subarachnoid space). The system may be used in the future as a tool for meningitis screening in newborns. An “optoacoustically active” CSF could be indicative of the presence of blood due to infection. Several potential clinical applications can be easily envisioned for the assessment and treatment of burns [29] or muscle diseases [30], among many others. Further work should explore the feasibility of all these applications.

Like every imaging method, the non-tomographic algorithm has its own imaging parameters and limitations. The axial resolution of the imaging method corresponds to the axial resolution of standard optoacoustic system, i.e., (*δ* = 0.88 $V_{s}/B$). Such a resolution figure holds if the imaged slab is ideal. Deviations from the ideal shape would result in a worse resolution figure since the algorithm cannot retrieve irregularities in the slab boundary. Also, the imaging method has no lateral resolution. Moreover, the algorithm might be prone to artifacts in the presence of highly absorbing non-planar structures or planar structures that are situated only partially below the detector.

Another limitation results from the fact that the algorithm might not work if the distance from the object to be imaged to the detector is sufficiently large. More in detail, the plane wave will keep its form indefinitely in time only if the slab has an infinite height. In the case of real-life finite slab, the wavefront will start to “degenerate” after traveling a certain distance from the source, becoming more spherical and destroying the core assumption of the algorithm. As a rule of thumb, one should avoid imaging beyond the Fresnel zone of the transducer.

While the simulations have focused on a homogeneous slab, the algorithm will work equally with non-homogenous slabs, provided that they keep a layered structure. Also, the simulated signals only show positive peaks as predicted by the theory [21]. The experimental signals instead display positive and negative peaks due to the finite bandwidth of the transducers among other factors. Our algorithm accounts for this through the Hilbert transform of the signals.

In its current form, the cost of the system is largely given by the cost of the Cobolt laser (EUR ~7k, Q-switched), delivering an energy per pulse of the order of tens of microjoules. However, the laser can be easily replaced by overdriven laser diodes [31,32] (EUR ~100 including the driver) with less energy per pulse (hundreds of nanojoules) at the expense of signal averaging. The fact that no scanning is performed allows us to perform extensive signal averaging. Overdriven laser diodes offer great versatility for signal averaging since their repetition rates can range from single shot up to MHz. On the other hand, the reconstruction algorithm requires minimal computational power, since no back-projection or model-based reconstruction is needed. Moreover, the cost of the transducer is EUR ~1 k, but generally, its price decreases significantly when ordered in sufficiently high volumes. Taking all this into account and assuming a cheap FPGA-based data acquisition system, the cost of the system can stay well below EUR 1k.

The method can be adapted to image structures with other shapes. To that end, the transducer detection surface must follow the shape of the structure and be placed parallel to its surface; moreover, the shape must follow certain symmetries. If these conditions are met, the individual waves emitted by each infinitesimal region of the structure (Huygens principle) will arrive in phase to the transducer surface, enhancing the signal. For example, a single detector following a line shape would allow us to image elongated structures like large blood vessels. Detection of vessels for venipuncture remains underdeveloped, being a long-standing unmet clinical need.

All in all, this work paves the way to achieve easy-to-use and ultra-low-cost optoacoustic imaging systems with the potential to improve the clinical routine in several medical fields.

## Data Availability

The raw data supporting the conclusions of this article will be made available by the authors on request.

## Abbreviations

The following abbreviations are used in this manuscript:

CSF	Cerebrospinal fluid
OAI	Optoacoustic imaging
ROI	Region of interest
RSOM	Raster-scan optoacoustic mesoscopy
US	Ultrasound

## References

[1] Spahn M. (2013). X-Ray Detectors in Medical Imaging. Nucl. Instrum. Methods Phys. Res. A.

[2] Mariani G., Bruselli L., Kuwert T., Kim E.E., Flotats A., Israel O., Dondi M., Watanabe N. (2010). A Review on the Clinical Uses of SPECT/CT. Eur. J. Nucl. Med. Mol. Imaging.

[3] Huang H., Wu R.S., Lin M., Xu S. (2024). Emerging Wearable Ultrasound Technology. IEEE Trans. Ultrason. Ferroelectr. Freq. Control.

[4] Attia A., Balasundaram G., Moothanchery M., Dinish U.S., Bi R., Ntziachristos V., Olivo M. (2019). A Review of Clinical Photoacoustic Imaging: Current and Future Trends. Photoacoustics.

[5] Omar M., Aguirre J., Ntziachristos V. (2019). Optoacoustic Mesoscopy for Biomedicine. Nat. Biomed. Eng..

[6] Aguirre J., Gasteiger C., Hindelang B., Seeger M., Bereznhoi A., Weidenfeld I., Darsow U., Stiel A.C., Steimle-Grauer S.A., Posch C. (2025). Non-Invasive Characterization of Melanoma Depth at Single-Cell Resolution. J. Eur. Acad. Dermatol. Venereol..

[7] Nau T., Schonmann C., Hindelang B., Riobo L., Doll A., Schneider S., Englert L., He H., Biedermann T., Darsow U. (2023). Raster-Scanning Optoacoustic Mesoscopy Biomarkers for Atopic Dermatitis Skin Lesions. Photoacoustics.

[8] Knieling F., Lee S., Ntziachristos V. (2025). A Primer on Current Status and Future Opportunities of Clinical Optoacoustic Imaging. npj Imaging.

[9] Deán-Ben X.L., Razansky D. (2021). Optoacoustic Imaging of the Skin. Exp. Dermatol..

[10] Assi H., Cao R., Castelino M., Cox B., Gilbert F.J., Gröhl J., Gurusamy K., Hacker L., Ivory A.M., Joseph J. (2023). A Review of a Strategic Roadmapping Exercise to Advance Clinical Translation of Photoacoustic Imaging: From Current Barriers to Future Adoption. Photoacoustics.

[11] Oraevsky A.A., Clingman B., Zalev J., Stavros A.T., Yang W.T., Parikh J.R. (2018). Clinical Optoacoustic Imaging Combined with Ultrasound for Coregistered Functional and Anatomical Mapping of Breast Tumors. Photoacoustics.

[12] Park J., Choi S., Knieling F., Clingman B., Bohndiek S., Wang L.V., Kim C. (2025). Clinical Translation of Photoacoustic Imaging. Nat. Rev. Bioeng..

[13] Hindelang B., Aguirre J., Schwarz M., Berezhnoi A., Eyerich K., Ntziachristos V., Biedermann T., Darsow U. (2018). Non-Invasive Imaging in Dermatology and the Unique Potential of Raster-Scan Optoacoustic Mesoscopy (RSOM). J. Eur. Acad. Dermatol. Venereol..

[14] Ntziachristos V. (2025). Addressing Unmet Clinical Need with Optoacoustic Imaging. Nat. Rev. Bioeng..

[15] Liu X., Villani F., Esteban I., Chang X., Zhang B., Cossettini A., Deán-Ben X.L., Benini L., Razansky D. (2025). Handheld Embedded Laser-Diode Illumination Optoacoustic System for Real-Time 3D Angiography of Deep Tissues. Laser Photonics Rev..

[16] Buehler A., Kacprowicz M., Taruttis A., Ntziachristos V. (2013). Real-Time Handheld Multispectral Optoacoustic Imaging. Opt. Lett..

[17] Zhang H., Maslov K., Stoica G., Wang L. (2006). Functional Photoacoustic Microscopy for High-Resolution and Noninvasive in Vivo Imaging. Nat. Biotechnol..

[18] Benavides Lara J., Prakash R., Avanaki K. (2025). Assessment of a Single-Element Scanning System for Enhanced Photoacoustic Imaging of Brain Hemorrhage. J. Biophotonics.

[19] Gao S., Tsumura R., Vang D.P., Bisland K., Xu K., Tsunoi Y., Zhang H.K. (2022). Acoustic-Resolution Photoacoustic Microscope Based on Compact and Low-Cost Delta Configuration Actuator. Ultrasonics.

[20] Han M., Lee Y.J., Ahn J., Nam S., Kim M., Park J., Ahn J., Ryu H., Seo Y., Park B. (2025). A Clinical Feasibility Study of a Photoacoustic Finder for Sentinel Lymph Node Biopsy in Breast Cancer Patients: A Prospective Cross-Sectional Study. Photoacoustics.

[21] Wang L.V., Wu H.-I. (2007). Biomedical Optics: Principles and Imaging.

[22] Guo Z., Li L., Wang L.V. (2009). On the Speckle-Free Nature of Photoacoustic Tomography. Med. Phys..

[23] Deán-Ben X.L., Razansky D. (2016). On the Link between the Speckle Free Nature of Optoacoustics and Visibility of Structures in Limited-View Tomography. Photoacoustics.

[24] Treeby B.E., Cox B.T. (2010). K-Wave: MATLAB Toolbox for the Simulation and Reconstruction of Photoacoustic Wave Fields. J. Biomed. Opt..

[25] Jaeger M., Schüpbach S., Gertsch A., Kitz M., Frenz M. (2007). Fourier Reconstruction in Optoacoustic Imaging Using Truncated Regularized Inverse K-Space Interpolation. Inverse Probl..

[26] Farkas J.P., Hoopman J.E., Kenkel J.M. (2013). Five Parameters You Must Understand to Master Control of Your Laser/Light-Based Devices. Aesthet. Surg. J..

[27] Berezhnoi A., Aguirre J., Hindelang B., Garzorz-Stark N., Omar M., Darsow U., Eyerich K., Ntziachristos V. (2019). Optical Features of Human Skin Revealed by Optoacoustic Mesoscopy in the Visible and Short-Wave Infrared Regions. Opt. Lett..

[28] van la Parra R.F.D., Deconinck C., Krug B. (2024). Diagnostic Imaging in Lipedema: A Systematic Review. Obes. Rev..

[29] Yamazaki M., Sato S., Ashida H., Saito D., Okada Y., Obara M. (2005). Measurement of Burn Depths in Rats Using Multiwavelength Photoacoustic Depth Profiling. J. Biomed. Opt..

[30] Tan L., Zschüntzsch J., Meyer S., Stobbe A., Bruex H., Regensburger A.P., Claßen M., Alves F., Jüngert J., Rother U. (2024). Non-Invasive Optoacoustic Imaging of Glycogen-Storage and Muscle Degeneration in Late-Onset Pompe Disease. Nat. Commun..

[31] Seeger M., Stylogiannis A., Prade L., Glasl S., Ntziachristos V. (2023). Overdriven Laser Diode Optoacoustic Microscopy. Sci. Rep..

[32] Stylogiannis A., Prade L., Buehler A., Aguirre J., Sergiadis G., Ntziachristos V. (2018). Continuous Wave Laser Diodes Enable Fast Optoacoustic Imaging. Photoacoustics.

